# Snow vole (*Chionomys nivalis* Martins) affects the redistribution of soil organic matter and hormone‐like activity in the alpine ecosystem: ecological implications

**DOI:** 10.1002/ece3.1727

**Published:** 2015-09-29

**Authors:** Diego Pizzeghello, Stefania Cocco, Ornella Francioso, Erika Ferrari, Alessandra Cardinali, Serenella Nardi, Alberto Agnelli, Giuseppe Corti

**Affiliations:** ^1^ Dipartimento di Agronomia, Animali, AlimentiRisorse Naturali e Ambiente (DAFNAE)Università di PadovaLegnaroPadovaItaly; ^2^ Dipartimento di Scienze Agrarie, Alimentari e AmbientaliUniversità Politecnica delle MarcheAnconaItaly; ^3^ Dipartimento di Scienze AgrarieUniversità di BolognaBolognaItaly; ^4^ Dipartimento di Scienze Chimiche e GeologicheUniversità di Modena e Reggio EmiliaModenaItaly; ^5^ Dipartimento di Scienze Agrarie, Alimentari ed AmbientaliUniversità di PerugiaPerugiaItaly

**Keywords:** ATR/FTIR, fulvic and humic acids, functional ecology, HRMAS‐NMR, Italian Long‐Term Ecological Research site, pedoturbation

## Abstract

In alpine environments, colonies of snow vole (*Chionomys nivalis* Martins) cause strong pedoturbation, which may affect humification process and soil organic matter (SOM) cycling, with repercussions on the hormone‐like activity of organics. We investigated the effect of snow vole pedoturbation on the chemical and spectroscopic features of soil organic fractions, and the potential hormone‐like activity of humic and fulvic acids (HA, FA). The study site was located on the high‐mountain environment of the Majella massif (central Italy). Pedoturbated and regular soils were morphologically described and characterized for pH and content of total organic carbon, total extractable carbon, HA, and FA. Both HA and FA were extracted and investigated using attenuated total reflectance/Fourier transform infrared (ATR/FTIR), nuclear magnetic resonance with high‐resolution magic angle spinning (HRMAS‐NMR), and ^1^H‐^13^C heteronuclear single quantum coherence (HSQC). HA and FA were also tested for their auxin‐like and gibberellin‐like activities. Results provide evidences that bioturbated and regular soils contain a poorly decomposed SOM, but HA and FA with a well‐defined molecular structure. The HA and FA from both bioturbated and regular soils show a hormone‐like activity with a different allocation along the soil profile. In the regular soil, the highest auxin‐like activity was shown by HA and FA from Oe1 horizon, while gibberellin‐like activity was expressed by FA from Oe2 horizon. Burrowing activity determines a redistribution of organics throughout the profile with a relatively high auxin‐like activity in the FA from straw tunnel wall (STW) and gibberellin‐like activity in the HA from vole feces (VF). The relative high presence of carboxylic acids, amides, proteins, and amino acids in the FA from STW and the aromatic moieties in the HA from VF put evidences for their different behavior. The fact that snow vole activity has modified the chemical and biological properties of SOM in these soils otherwise considered governed only by low temperature has important ecological implications such as the preservation of soil fertility and vegetal biodiversity.

## Introduction

Alpine soils cover about 4 × 10^6^ km^2^ worldwide and are generally characterized by high altitudes and slope, with rocky and stony features and sparse vegetation. In Italy, alpine soils are diffused at elevated altitudes in the Alps and the Apennines. On the Apennines, a mountain range consisting of parallel chains extending about 1500 km North to South, the Majella massif has caught our interest for the abundance of kettle holes at the bottom of the principal valleys (Corti et al. 2012). The areas dotted by kettle holes host a lot of colonies of snow vole (*Chionomys nivalis* Martins), a little fossorial mammal.

Vole is one of the main pedoturbators of recently deglaciated areas or extreme cold and dry environments (Wilkinson et al. 2009) and is able to produce surface mounds, soil burial, and downslope transport (Zaitlin and Hayashi 2012). Consequently, this small mammal affects pedogenesis by mixing soil horizons and modifying water holding capacity, nutrient status, and biological activity. Further, as the snow vole is a glacial relict, and Würm glaciers on the massifs of central Italy vanished about 11,000 years before present, we suspected that, during millennia, the vole has been harmonizing with the environment also by fostering species it needs for its own survival and that this animal–plant relationship may have a biochemical explanation.

Alpine soils have received increasing attention in recent years (Geng et al. 2012) as they are able to stock soil organic matter (SOM) in critical environmental conditions where low temperatures and low turnover rates of soil organic carbon are expected (Celi et al. 2010; Zhuang et al. 2010; Budge et al. 2011; DeLuca and Zabinski 2011; Glanville et al. 2012). Despite this, there is still insufficient information on the role of bioturbation on SOM cycling and properties in alpine soils. In this study, the soil from home ranges of *C. nivalis* in the high‐mountain environment of the Majella massif (Italy) was morphologically described and analyzed. Regular (not bioturbated) soils were also described and analyzed. Total extractable carbon was quantified, while humic acids (HA) and fulvic acids (FA) were separated and characterized to assess their structure and hormone‐like activity. The objectives of this study were to assess (1) the changes induced by *C. nivalis* on soil properties, (2) SOM properties and biological activity, and (3) the ecological implications of both the above‐mentioned factors.

## Materials and Methods

### Study site

**Figure 1:**
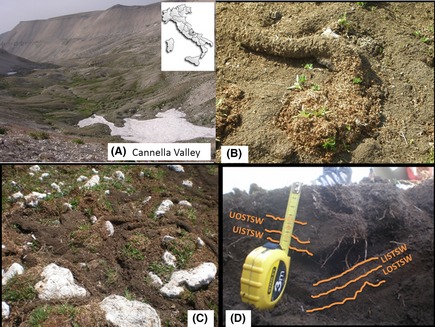
(A) View of the Cannella Valley, Central Apennines, with the bottom valley pitted of kettle holes hosting *Chionomys nivalis* home ranges; inset, position of the Valley in Italy. (B) The rests of the vegetables (straw) that lined the snow season galleries, with sprouts of recently germinated seeds; seeds took advantage by the mulching effect induced by the straw pile, while sprouts took advantage by the stimulation due to hormone‐like activity of the humic substances. (C) Arrangement of the different type of superficial galleries. (D) Burrow organization with indicated the different materials sampled (for abbreviations see text).

The Majella massif is one of the Italian Long‐Term Ecological Research sites and is located on Central Apennines, Italy (Fig. 1A). The massif is dominated by 30 summits in excess of 2000 m above sea level and shows geomorphological features of past glacial conditions. Much information on physiography and geology of the massif is reported in Corti et al. (2012). Here, it suffices to report that the main glacial valleys are covered by thick morainic deposits (calcareous rocks) that form closely associated small mounds (*kames*) with interspersed depressions (*kettle holes*). The principal valley, the Cannella Valley, is about 5 km long and from 1 to 1.5 km wide, spans from 1900 and 2750 m of altitude, and represents the site of this study (Fig. 1A). Over an altitude of 2000 m, there are periglacial conditions with a mean annual air temperature of 2.3°C (January is the coldest month [−4.3°C] and August the warmest one [11.4°C]), and a mean annual precipitation of about 2100 mm, including snow water equivalent. In normal years, snow cover is large, from mid‐September to mid‐June.

On the reliefs of the deposits and on the flanks of the valley, there are skeletal or very skeletal Entisols (Soil Survey Staff 2010) and the stacking of the clasts fosters a drainage that induces summer *xeric* conditions. Because of this, these soils generally have a scarce vegetal cover (5–20%). In contrast, soils inside kettle holes are Mollisols (Soil Survey Staff 2010) often rocky or skeletal as well, but the clasts are immersed in a silty and SOM‐rich earthy matrix. Owing to their physiographic position and the contents of fine earth and SOM, these soils retain more water and, consequently, are completely grass covered except for large blocks that may be present at site. The rather severe climatic conditions selected flora and fauna species. The arboreal vegetation is absent and there are few shrub species considered as glacial relicts: *Salix retusa* L., *Silene acaulis* (L.) Jacq., and *Saxifraga oppositifolia* subsp. *speciosa* (Dörfl. & Hayek) Engl. & Irmsh.. There are also some herbaceous species such as *Trifolium thalii* Vill., *Taraxacum apenninum* (Ten.) DC., *Crepis aurea* subsp. *glabrescens* (Caruel) Arcang., *Phleum alpinum* subsp. *rhaeticum* Humpries, and *Plantago atrata* Hoppe.

### The snow vole

The snow vole *Chionomys nivalis* Martins is a little mammal spread on the mountain chains of Europe and Asia that prefers skeletal and rocky soils. In Italy, this vole preferably occupies areas at heights comprised from 1000 to 2500 m, so it abounds on the Alps, northern Apennines and, more south, on Gran Sasso and Majella massifs (Spagnesi and De Marinis 2002). In the Mediterranean basin, Majella massif is one of the southernmost site hosting snow vole colonies, and the territory interested by colonies accounts for several km^2^.

The snow vole has a head‐body length of 90–140 mm, and a tail length of 47–75 mm. It has a strictly vegetarian diet that, on the basis of the plant species, comprises stems, leaves, flowers, seeds, and roots (Spagnesi and De Marinis 2002). The ecology of this species and its altitudinal distribution is mainly due to stable stenothermic soil conditions, soil stoniness (Nappi 2002), and snow cover duration. In summer, each colony produces a home range (Burt 1943), usually reworking that of the year before. The home range occupies a surface of some dozen of square meters and includes a burrowing system made of galleries and several chambers that hosts the voles. During the cold season, soil freezes and the vole excavates galleries and chambers at the soil–snow interface, where it remains active for the entire period of snow. The winter galleries and chambers are lined with vegetables so to obtain a proper insulation. One of these chambers hosts the nest, whereas one or more are used to stock dry vegetables and one is for feces. After snow smelt, the rests of the lining vegetables lie at the soil surface together with the rests of the summer superficial galleries (Fig. 1B and C).

### Field operations

During summers 2010, 2011 and 2012, many snow vole home ranges inside kettle holes were examined. In summer 2012, three of the monitored home ranges were transected to observe the convolution of the underground burrowing. Each home range was contrasted with a regular (not bioturbated) soil coming from an adjacent kettle hole that was similarly transected. Bioturbated and regular soils were morphologically described per Schoeneberger et al. (2002). At the surface of the bioturbated soil, we identified the following remainders of the snow tunnels (Fig. 1B and C): straw of the tunnel walls (STW), light‐colored material of the tunnel walls (LTW), brown‐colored material of the tunnel walls (BTW), and piles of vole feces that were accumulated in the snow chamber (VF). For the soil galleries (Fig. 1D), within the A2 and A2′ soil horizons, a 2‐cm‐thick inner layer was separated from a 2‐cm outer layer, so to obtain four types of sample per each gallery: upper‐outer layer of the soil tunnel wall (UOSTW), upper‐inner layer of the soil tunnel wall (UISTW), lower‐inner layer of the soil tunnel wall (LISTW), and lower‐outer layer of the soil tunnel wall (LOSTW). Regular soil was made of a sequence of not bioturbated horizons. All these materials were sampled, stored in a portable refrigerator, and transported to the laboratory.

### Laboratory analysis

**Figure A1:**
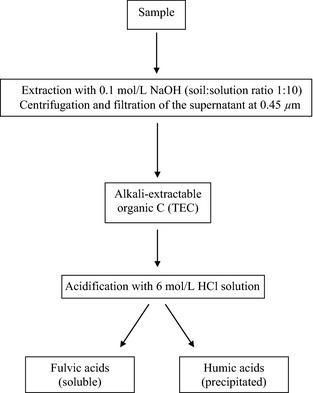
Scheme of the extraction and fractionation of humic and fulvic acids.

The samples were air‐dried. The proportion of soil skeleton was determined by weight after sieving the bulk samples at 2 mm. The pH was carried out in H_2_O (1:2.5, w/v). The total organic carbon (TOC) content was determined by dichromate digestion, heating the suspension at 180°C for 30 min (Allison 1965). Total extractable C (TEC) was obtained by a 0.1 mol/L NaOH solution (Stevenson 1994) and, from this extract, HA and FA were separated according to the procedure of Swift (1996), freeze‐dried, and analyzed for their C content (Allison 1965). Details about separation of HA and FA are reported in Appendix (Fig. A1).

The structure of the extracted HA and FA was investigated by attenuated total reflectance/Fourier transform infrared (ATR/FTIR), nuclear magnetic resonance with high‐resolution magic angle spinning (HRMAS‐NMR), and ^1^H‐^13^C heteronuclear single quantum coherence (HSQC).

In more detail, FTIR spectra were recorded on solid samples using a Nicolet 5700 Thermo‐Corporation equipped with a diamond attenuated total reflectance (ATR) accessory (Spectra‐Tech, Shelton, CT), by co‐addition of 100 scans at a resolution of 4 cm^−1^ in the range 4000–400 cm^−1^. A background spectrum using the diamond crystal was only recorded prior to collection of each sample spectrum. The second derivative of the IR spectra in the 1850–1150 cm^−1^ region was used to define some starting parameters for the curve fitting analysis, such as the number and position of peak components by the Grams/386 spectroscopic software (version 6.00, Galactic Industries Corporation, Salem, NH). The IR spectra were fitted using Gaussian shape bands. The optimum Gaussian curve fitting was determined by the lowest value for the minimization function of reduced chi‐square (*χ* ^2^) and a good agreement between experimental and calculated profiles [coefficients of determination (*R* ^2^) between 0.999 and 0.988, standard error in the range 0.001–0.003].

For HRMAS‐NMR, each sample (~30 mg) was dissolved in 100 *μ*L of deuterated dimethyl sulfoxide (DMSO‐d_6_), properly homogenized, then introduced in a 90‐*μ*L HRMAS zirconium rotor (4 mm OD type) and transferred into the MAS probe. HRMAS‐NMR spectra were recorded with a Bruker FT‐NMR Avance 400 Spectrometer at 25°C using 8 kHz spinning rate. Nominal frequencies were 400.13 MHz for ^1^H and 100.61 MHz for ^13^C. An internal lock on the deuterium of DMSO‐d_6_ was used for all spectra. The chemical shifts were referred to tetramethylsilane (TMS). One‐dimensional NMR data were acquired using standard pulse, bipolar longitudinal eddy current delay pulse (BPLED), and Carr–Purcell–Meiboom‐Gill (CPMG) sequences, commonly known as “zg”, “ledbpgs2s,” and “cpmg1d” in the standard Bruker library, respectively. As regards, the BPLED sequence, 4000 scans were collected, implying a bipolar pulse pair ranging from 2 to 3 ms, with a diffusion time of 100–200 ms, and a time domain point of 8000–16,000. The gradient length and diffusion time were varied in each sample in order to achieve 95% signal suppression at maximum gradient strength. For the one‐dimensional CPMG acquisitions, in order to eliminate diffusion and J effects, the ECHO evolution delay was set to 1 ms, the number of loops (L1) ranged from 80 to 180, scans were 4000, time domain points 16,000–32,000, and relaxation delay was 2 sec. ^1^H NMR spectra were integrated using the software Amix 3.7.10 (Bruker Biospin GMBH, Rheinstetten, Germany). For each sample, the integration was performed on specific spectral regions. Each integrated area is the average value of three independent calculations; standard error was <5%.

The hormone‐like activity of both HA and FA was assessed for their auxin‐ and gibberellin‐like activity. Specifically, the auxin‐like activity was estimated by measuring the reduction in the growth of watercress (*Lepidium sativum* L.) roots after the application of hormone (indole‐3‐acetic acid, IAA) and humic substances, while the gibberellin‐like activity was determined by the increase in the length of lettuce (*Lactuca sativa* L.) epicotyls after the application of hormone (gibberellic acid, GA) and humic substances. Both these bioassays are typically used for assessing auxin‐ and gibberellin‐like activities (Audus 1972; Pizzeghello et al. 2002). In detail, watercress and lettuce seeds were surface‐sterilized by immersion in 8% hydrogen peroxide for 15 min. After rinsing five times with sterile distilled water, 10 seeds were placed on a sterile filter paper in a sterile Petri dish. For watercress, the filter paper was wetted with 1.2 mL of a 1 mmol/L CaSO_4_ solution (control), or 1.2 mL of 20, 10, 1, and 0.1 mg L^−1^ IAA solution (Sigma, StLouis, MO) for the calibration curve, or 1.2 mL of 10, 5, 2.5, 1, 0.5, 0.2, and 0.1 mg L^−1^ of C as HA or FA solution. For lettuce, the experimental design was the same as for watercress except that the sterile filter paper was wetted with 1.4 mL instead of 1.2 mL, and the calibration curve was a progression of 100, 10, 1, and 0.1 mg L^−1^ GA solution (Sigma). The seeds were germinated in the dark at 25°C in a germination room. After 48 h for watercress and 72 h for lettuce, the seedlings were removed and the root or epicotyl lengths measured with a TESA‐CAL IP67 electronic caliper (TESA, Renens, CH) and Data Direct software, version 1 (ArtWare, Asti, IT). Data were transformed on natural logarithmic scale to obtain the best linear fitting.

### Statistical analyses

All examined variables were tested for normality and homoscedasticity (by Shapiro–Wilk's and Levene's tests, respectively) and transformed when necessary to satisfy assumptions required by parametric statistics. Data were the means of three independent replicates, and the standard deviations did not exceed 5%. The Student–Newman–Keuls test was applied to compare the differences between group means (Sokal and Rohlf 1969), and regression analysis was used to calculate the HA and FA dose–response curves. The statistic was performed using SPSS for Windows software, version 18.0 (SPSS, Chicago, IL).

## Results

### Morphological features, pH, and organic carbon content of the soil profiles

Morphology of bioturbated and regular soils are reported in Table 1. The regular soil was uniformly vegetated by grasses. The two superficial horizons were organic (Oe1 and Oe2), with a well‐developed granular structure. The underlying A horizon displayed a well‐developed platy structure. All these horizons abounded of micro‐, very fine, fine, and medium roots and contained 10–20% calcareous skeleton. The bioturbated soil appeared rather barren, with seedlings emerging from the STW, LTW, and BTW materials; however, only the two latter materials contained abundant grass roots. All these three materials showed a weak to moderate state of aggregation and a very soft consistency. All the horizons forming the vertical section of the home range contained abundant micro‐, very fine, and fine roots. The skeleton (calcareous) was scarce in both the above‐ground materials (2–3%) and into the soil (5%). In spite of the calcareous nature of the parent rock, soil pH was always in the acidic field (Table 2). As expected, in the regular soil, pH increased with depth, while in the bioturbated soil, it was highest in the materials laying on the soil, decreased with depth to reach the lowest values in the tunnel vault, to increase more in depth.

**Table 1:** Morphological description of two representative profiles from adjacent kettle holes: “regular soil” is from an area not interested by the *Chionomys nivalis* home range, “bioturbated soil” is in the middle of the *Chionomys nivalis* home range. Cannella Valley, Majella massif, Italy

	Depth (cm)	Color^a^	Structure^b^	Consistence^c^	Roots^d^	Skeleton		Other observations
						Content^e^	Size	
						%	cm	
Regular soil (Oxyaquic Haplocryoll, loamy–skeletal, mixed, frigid (Soil Survey Staff 2010)								
Oe1	21–13	10YR 3/2	3f g	m fr, w ss	3 mi, vf, f, m	10	1–15	No effervescence
Oe2	13–0	7.5YR 3/2	3f, m, c g	m fr, w ss	3 mi, vf, f, m	10	1–15	No effervescence
A	0–50	7.5YR 3/1	3th, m pl	m fr, w ss	2 mi, vf, f, m	20	7–15	No effervescence
Bioturbated soil (Oxyaquic Haplocryoll, loamy–skeletal, mixed, frigid (Soil Survey Staff 2010)								
Straw tunnel wall (STW) (O material)	7–0	10YR 2/2	1 m g	–	–	2	0.1–0.2	No effervescence, very soft consistency, seedlings
Vole feces (VF) (O material)	3–0	10R 2.5/2	3 m g	–	–	0	–	No effervescence
Light tunnel wall (LTW) (A material)	8–0	7.5YR 2.5/3	2 m g	m fr, w s	3 mi, vf, f	3	0.1–0.2	No effervescence; very soft consistency, seedlings
Brown tunnel wall (BTW) (A material)	8–0	5YR 2.5/2	2 m g	m fr, w s	3 mi, vf, f	3	0.1–0.2	No effervescence, very soft consistency, seedlings
A1	0–4	5YR 3/3	3f g	m fr, w s	3 mi, vf, f	5	0.3–0.5	No effervescence
Upper–outer layer of the soil tunnel wall (UOSTW) (A2)	4–6	10YR 2/2	3f g	m fr, w s	3 mi, vf, f	5	0.3–0.5	No effervescence
Upper–inner layer of the soil tunnel wall (UISTW) (A2)	6–8	5YR 2.5/1	3f g	m fr, w s	3 mi, vf, f	5	0.3–0.5	No effervescence
Tunnel	8–13	–	–	–	–	–	–	Straw and seeds
Lower–inner layer of the soil tunnel wall (LISTW) (A2′)	13–15	5YR 2.5/1	3f g	m fr, w s	3 mi, vf, f	5	0.3–0.5	No effervescence
Lower–outer layer of the soil tunnel wall (LOSTW) (A2′)	15–17	10YR 2/2	3f g	m fr, w s	3 mi, vf, f	5	0.3–0.5	No effervescence
A3	17–75	7.5YR 2.5/1	3f, m g	m fr, w s–ss	3 mi, vf, f	5	0.3–0.5	No effervescence

a Moist and crushed, according to the Munsell Soil Color Charts (1992 edition).

b 1 = weak, 2 = moderate, 3 = strong; f, fine; m, medium; c, coarse; th, thin; g, granular; pl, platy.

c m, moist; fr, friable; w, wet; ss, slightly sticky; s, sticky.

d 2 = plentiful, 3 = abundant; mi, micro; vf, very fine; f, fine; m, medium.

e By mass.

**Table 2:** Contents of total organic carbon (TOC), total extractable carbon (TEC), humic acids (HA), fulvic acids (FA), and TEC/TOC and FA/TEC ratios in regular and *Chionomys nivalis* bioturbated soils^a^. Cannella Valley, Majella massif, Italy

	Depth, cm	pH	TOC	TEC	HA	FA	TEC/TOC	FA/TEC
			g C kg^−1^				%	
Regular soil								
Oe1	21–13	5.11d	440.12a	74.29a	63.70a	10.59b	16.9cd	14.3c
Oe2	13–0	5.23d	280.15b	58.52b	49.26b	9.26b	20.9c	15.8c
A	0–50	5.78c	77.16d	14.55d	12.31d	2.24d	18.9c	15.4c
Bioturbated soil								
Straw tunnel wall STW) (O material)	7–0	6.18bc	294.60b	17.06d	10.89d	6.17c	5.8e	36.2b
Vole feces (VF) (O material)	3–0	6.35b	376.61b	48.49c	21.98c	26.51a	12.9d	54.7a
Light tunnel wall (LTW) (A material)	8–0	6.84a	64.79d	9.43e	3.88f	5.55c	14.4d	59.4a
Brown tunnel wall (BTW) (A material)	8–0	6.31b	91.17c	16.99d	10.73d	6.26c	18.6c	36.8b
A1	0–4	5.47 cd	79.32d	14.40d	nd	nd	18.2c	nd
Upper–outer layer of the soil tunnel wall (UOSTW) (A2)	4–6	4.87e	24.05f	7.24f	3.60f	3.64c	30.1b	50.3a
Upper–inner layer of the soil tunnel wall (UISTW) (A2)	6–8	4.66f	28.03e	8.89e	4.13f	4.76c	31.7b	53.5a
Lower–inner layer of the soil tunnel wall (LISTW) (A2′)	13–15	5.30d	34.35e	12.67d	6.74e	5.93c	36.9a	46.8a
Lower–outer layer of the soil tunnel wall (LOSTW) (A2′)	15–17	5.19d	31.34e	9.76e	4.69f	5.07c	31.1b	51.9a
A3	17–75	5.58 cd	29.62e	9.89e	nd	nd	33.4ab	nd

nd, not determined.

In each column, mean values with different letters significantly differ for *P* ≤ 0.05 by Student–Newman–Keuls test.

a Mean values of the three regular (not bioturbated) and three bioturbated soils.

In general, TOC, TEC, HA, and FA contents of regular soil were higher than those of the bioturbated soil (Table 2). The only exceptions were VF and STW, which exhibited a TOC content similar to that of the Oe2 horizon of the regular soil. VF also exhibited the highest content of FA. In the regular soil, the percent of TEC over TOC showed a rather narrow range (16.9–20.9%), whereas it displayed a wider range in the bioturbated soil: from 5.8 to 18.6% in the above‐ground tunnel walls and in the A1 horizon, and from 30.1 to 36.9% more in depth. Moreover, in the bioturbated soil, the FA percentages over TEC were 2.3–3.8 fold higher than in the regular soil.

### Spectroscopic characteristics of HA and FA

**Figure 2:**
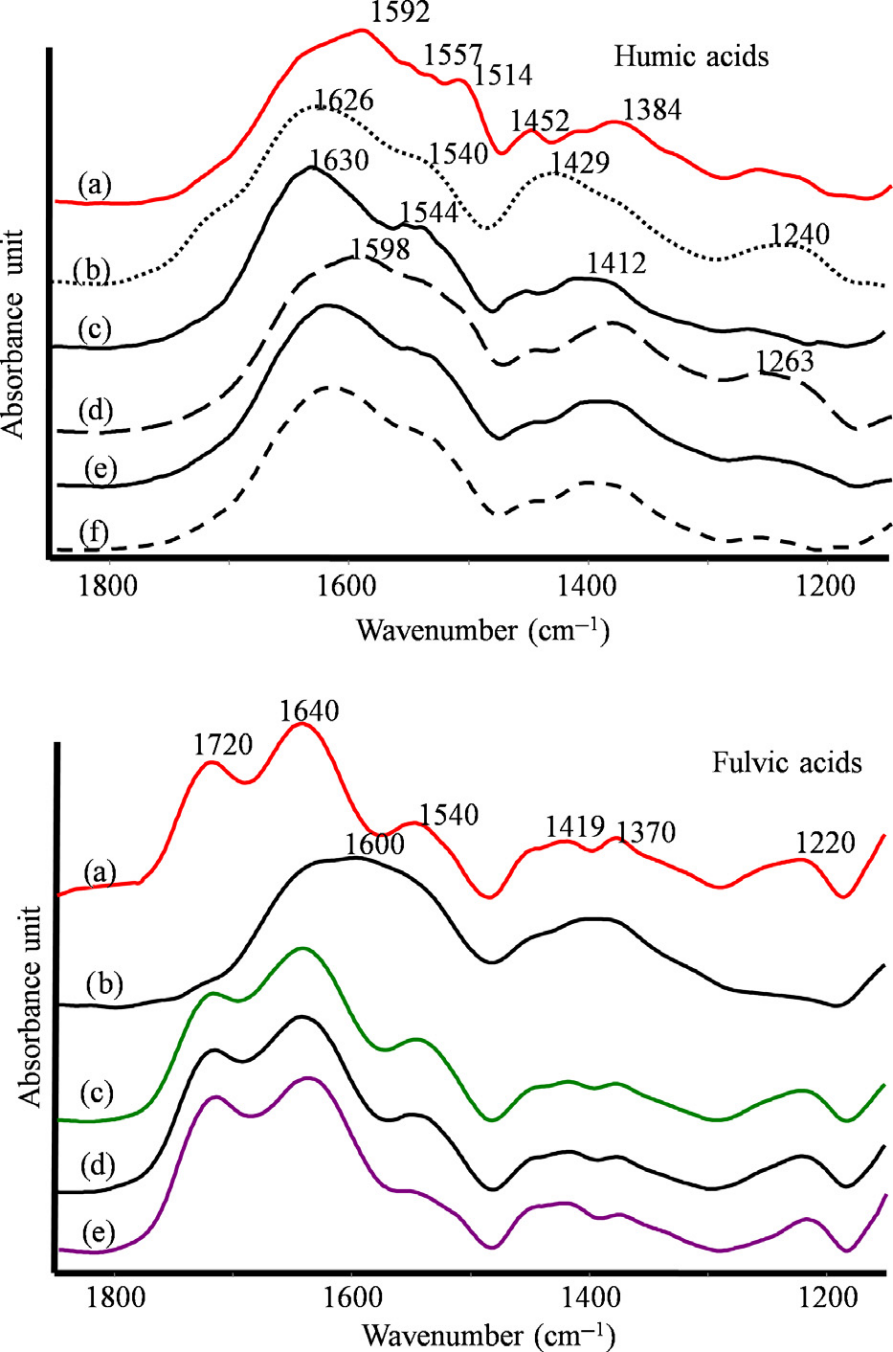
Attenuated total reflectance/Fourier transform infrared spectra of HA (upper) and FA (bottom) extracted from (a) vole feces (VF), (b) Oe2 horizon of regular soil, (c) light‐colored material of the superficial tunnel walls (LTW), (d) brown‐colored material of the superficial tunnel walls (BTW), (e) straw tunnel wall (STW), and (f) upper‐outer layer of the soil tunnel wall (UOSTW).

The ATR/FTIR spectra of HA and FA are shown in Figure 2. The region at high frequencies between 3400 and 2500 cm^−1^ did not give structural information and, therefore, it is not shown and discussed. The band around 1720 cm^−1^, due to C=O stretching of carboxylic acid (Stevenson 1994), appeared only in FA of the bioturbated soil materials, while it was a small shoulder in the FA from the Oe2 horizon of the regular soil and in the HA of both types of soil. A strong band at ~1640 cm^−1^, assigned to C=O stretching of amide I, quinones, and ketones (Mayo et al. 2004), and to C=C stretching vibration in olefins, was present in all FA and as a shoulder in the HA of VF and LTW. HA were characterized by a strong band at ~1620 and 1590 cm^−1^ that was ascribed to asymmetric COO^‐^ stretching vibration, while symmetric COO^−^ stretching motion appeared as a broad peak at around 1400–1380 cm^−1^ (Mayo et al. 2004). The bands at 1557–1554 cm^−1^ were ascribed to amide II and appeared in all spectra. The aromatic ring vibrations at 1538–1514 cm^−1^ were displayed in HA and FA of VF, and in HA from different tunnels. Other bands at 1446–1434 cm^−1^ were assigned to C‐H bending (CH_3_‐C and/or CH_2_‐), and the bands from 1378 to 1350 cm^−1^ were in the range of in‐plane bending of O–H (1350 ± 50 cm^−1^) and the C–H symmetric bending/umbrella mode (1375 ± 10 cm^−1^) (Mayo et al. 2004). The band at 1250 cm^−1^ was assigned to amide III, but also to C–O stretching and O–H bending of COOH groups and C‐O stretch in aryl ethers, while the peak at 1230 cm^−1^ was due to the C–OH stretching of phenols and C‐O symmetric stretching of aryl ethers.

**Figure 3:**
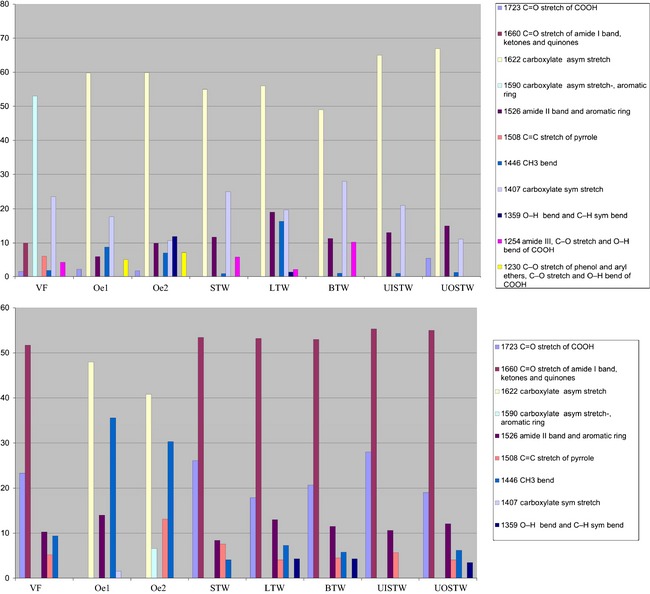
Histograms of HA (upper) and FA (lower) of the ATR/FTIR peak areas processed by Gaussian curve fitting.

More quantitative information on the ATR/FTIR spectra of HA and FA was obtained by Gaussian curve fitting procedure applied to the investigated region. The percentage area of each functional group can be considered representative of the HA and FA structures (Fig. 3). In detail, carboxylate groups represented the most abundant functional groups in HA. These groups accounted for 70% in the HA of UOSTW, 65% in UISTW, 60% in Oe1 and Oe2 horizons, 56% in LTW, 55% in STW, and 49% in BTW. In contrast, carboxylate groups in FA from regular soil accounted for 48 and 40% in Oe1 and Oe2, respectively. Carbonyl groups in HA accounted for 5.4% in UOSTW and 2.5% and 1.7% in Oe1 and Oe2, respectively. These groups were more abundant in FA, where they accounted for 28% in UISTW, 26% in STW, 20% in BTW, and 19 and 18% in UOSTW and LTW, respectively. The presence of amide I was the most representative in FA of the bioturbated soil, accounting for 55% in UISTW and UOSTW and 53% in STW, LTW, and BTW. Amide I was absent in FA of the regular soil. Aromatic rings were the second functional group that characterized HA; they accounted for 19% in LTW, 15% in UOSTW, 13% in UISTW, 12% in STW, 11% in BTW, and 9.9 and 6% in Oe1 and Oe2, respectively. In FA, the aromatic component might be influenced by the amide II contribution and, for this reason, we have considered all together the percentage area: 14% in Oe1, 13% in LTW, 12% in UOSTW, 11% in BTW, 10% in UISTW, 9% in Oe2, and 8% in STW. A minor influence was due to aliphatic groups (CH_3_), which in HA accounted for 16% in LTW, and for 9 in Oe1, 7% in Oe2, 1.3% in UOSTW, 1% in both BTW and UISTW, and 0.9% in STW. In FA, they accounted for 13% in Oe2, 7.6% in STW, 6% in UISTW, and 4% in LTW, BTW, and UOSTW. Similarly, C‐OH groups in HA accounted for 10% in BTW, 6% in STW, and 2.1% in LTW; 7 and 5% in Oe2 and Oe1, respectively. In FA, they accounted for 4% in both LTW and BTW, and 3.5% in UOSTW.

**Figure 4:**
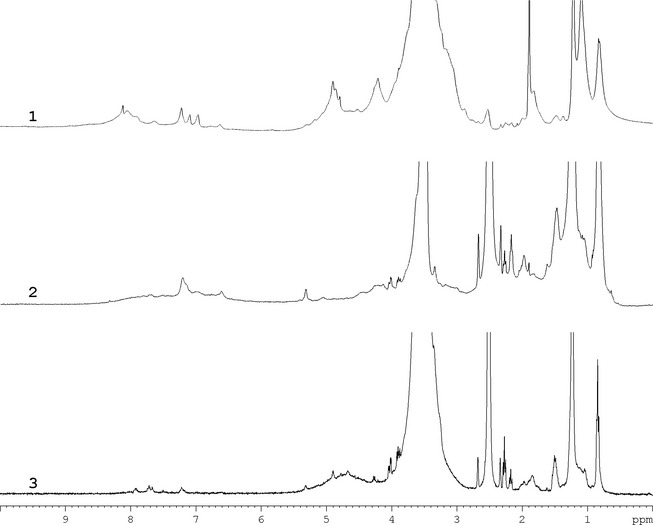
^1^H HRMAS‐NMR spectra of (1) FA from straw tunnel wall (STW), (2) HA from the Oe1 horizon of regular soil, and (3) FA from the Oe1 horizon of regular soil.

**Figure 5:**
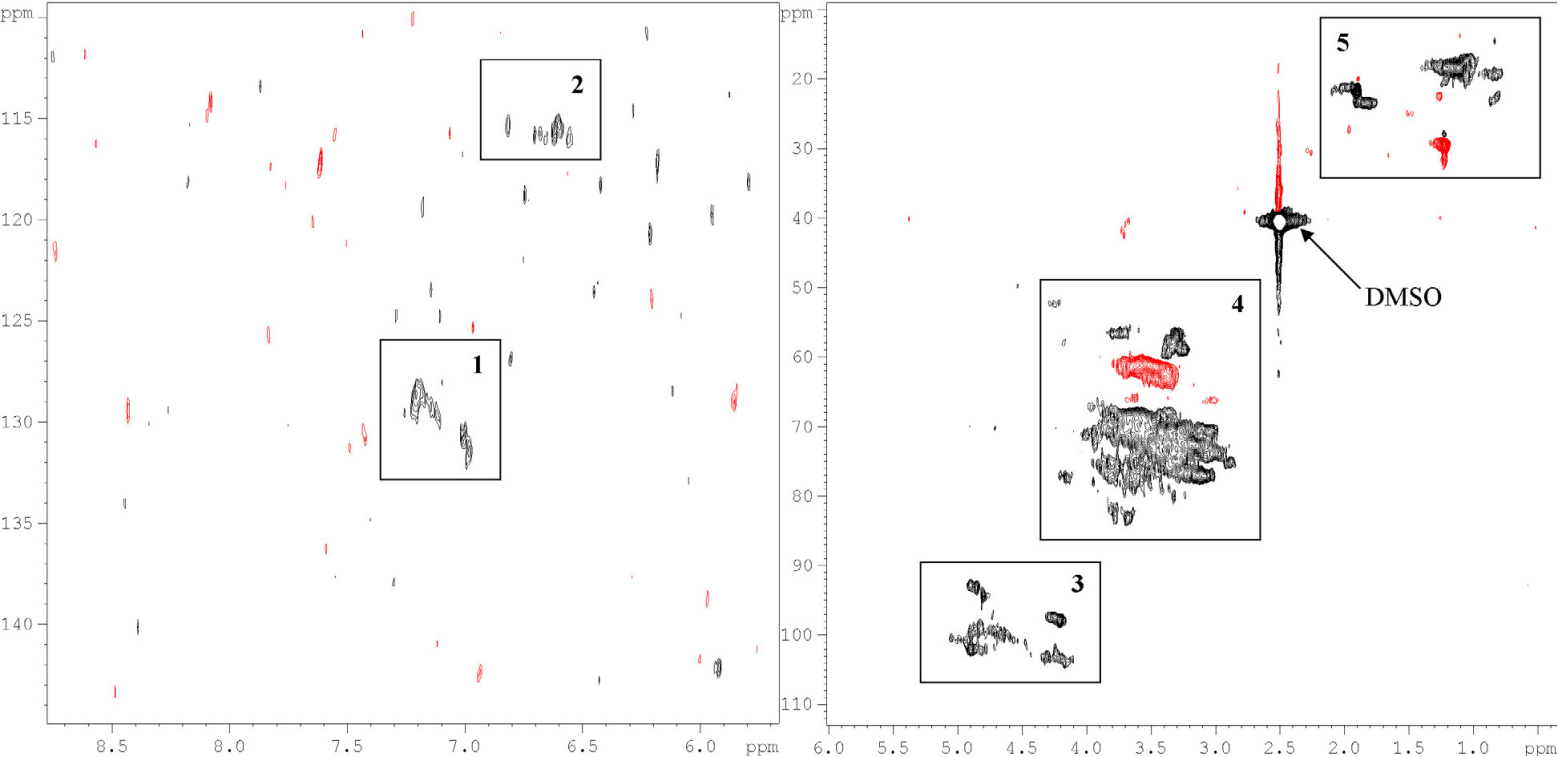
Phase‐sensitive ^1^H‐^13^C HSQC spectra of FA from straw tunnel walls (STW) of bioturbated soil. CH/CH_3_ and CH_2_ cross peaks are reported in black and red color, respectively. Typical assignments are reported by square boxes: (1) aromatic systems; (2) double bonds of conjugated systems; (3) anomeric correlations of sugar‐like species; (4) sugar‐like and peptide correlations, lignin side‐chains, aromatic methoxyl groups, and *α*‐protons from amino acids/peptides/proteins; (5) long and branched aliphatic chains from lipids and waxes.

Among the ^1^H HRMAS‐NMR spectra, we report those of HA and FA from the Oe1 horizon of the regular soil, and that of FA from STW (Fig. 4); these samples were those with the highest organic carbon content and hormone‐like activity (see later). In the Oe1 horizon of the regular soil, major differences between HA and FA were found in the aromatic region (6.5–8 ppm), which indicated a higher content of carboxylic groups in FA than in HA. This observation was confirmed by ^13^C HRMAS‐NMR spectrum (data not shown). In addition, FA were characterized by a relevant amount of carbohydrates (i.e., cellulose), peptides, and proteins, while HA were richer in aliphatic structures, mainly attributable to lipids and waxes. The ^1^H HRMAS‐NMR spectrum of FA from STW differed from that of FA of regular soil for the greatest amount of aromatic moieties with respect to aliphatic ones, and an increased complexity in the region at 4–5 ppm, mainly due to the presence of proteins. In Figure 5 ^1^H‐^13^C HSQC spectrum confirms the presence of aromatic signals with typical cross peaks highlighted by square box 1, together with correlations due to CH of *α*‐protons in peptides, proteins, and amino acids as well as carbohydrates such as cellulose.

### Hormone‐like properties of HA and FA

For the auxin‐like activity (Table 3), similar dose‐dependent responses were obtained with IAA (*P *≤* *0.001), HA from VF, LTW, BTW, and UISTW (*P *≤* *0.05), and FA from STW, BTW, and UISTW (*P *≤* *0.05). Significant responses (*P *≤* *0.01) were also induced by both HA and FA of the Oe1 horizon of the regular soil. In all cases, a logarithmic model explained a high percentage of the total variability of the dependent variable (*R* ^2 ^= 83–98%). In addition, the high values of correlation coefficient (*r* = 74–86%) indicated that the variables were closely related to each other. Thus, HA of the Oe1 horizon, FA of BTW, and HA of BTW had the highest values of both *R* ^2^ and *r*, whereas HA of the Oe1 horizon had also the highest *b* value among the regression curves. Notably, FA of STW and UISTW, and HA of LTW had *b* values between HA and FA of the Oe1 horizon.

**Table 3:** Coefficients of correlation and parameters of the regression curves [*Y* = *a* + *b*•log(*X*)] between concentration and root length of watercress plantlets treated with indole‐3‐acetic acid (IAA) or with the humic and fulvic acids (HA, FA) extracted from the regular and bioturbated soils. Cannella Valley, Majella massif, Italy

Treatment		*r*	*R* ^2^	df	*b*
IAA		0.821**	0.941***	235	−1.318
Regular soil					
Oe1	HA	0.845**	0.976***	172	−0.322
	FA	0.821**	0.953**	156	−0.236
Oe2	HA	n.s.	n.s.		n.s.
	FA	n.s.	n.s.		n.s.
Bioturbated soil					
Straw tunnel wall (STW)	HA	n.s.	n.s.		n.s.
	FA	0.766**	0.852**	161	−0.296
Vole feces (VF)	HA	0.762**	0.828***	180	−0.239
	FA	n.s.	n.s.		n.s.
Light tunnel wall (LTW)	HA	0.787**	0.837***	188	−0.268
	FA	n.s.	n.s.		n.s.
Brown tunnel wall (BTW)	HA	0.801**	0.98*	190	−0.149
	FA	0.858**	0.977*	158	−0.223
Upper‐outer layer of soil tunnel wall (UOSTW) (A2)	HA	n.s.	n.s.		n.s.
	FA	n.s.	n.s.		n.s.
Upper‐inner layer of soil tunnel wall (UISTW) (A2)	HA	0.788**	0.891*	175	−0.148
	FA	0.745**	0.827*	167	−0.266
Lower‐inner layer of soil tunnel wall (LISTW) (A2′)	HA	n.s.	n.s.		n.s.
	FA	n.s.	n.s.		n.s.
Lower‐outer layer of soil tunnel wall (LOSTW) (A2′)	HA	n.s.	n.s.		n.s.
	FA	n.s.	n.s.		n.s.

df, degree of freedom; n.s., not significant, meaning that no significant coefficient of correlation and regression was found.

*P ≤ 0.05; **P ≤ 0.01; ***P = 0.001.

For the gibberellin‐like activity (Table 4), significant was the dose‐dependent response induced by GA (*P *≤* *0.01). A reliable significance (*P *≤* *0.05) was also obtained for HA of VF, LTW, BTW, and UISTW, and for FA of STW, VF, UOLTW, and UISTW. For the regular soil, the same *P *≤* *0.05 significance was obtained for HA of the Oe1 and Oe2 horizons, and for FA of the Oe2 horizon. The curves fitted a logarithmic model and explained a high proportion of the total variability of the dependent variable (*R* ^2 ^= 87–99%); closely related between them were the variables (*r* = 72–90%). Among HA and FA, those of VF had the highest values of both *R* ^2^ and *r*, while FA of Oe2 horizon of regular soil had the highest *b* value.

**Table 4:** Coefficients of correlation and parameters of the regression curves [*Y* = *a* + *b*•log(*X*)] between concentration and steam length of lettuce plantlets treated with gibberellic acid (GA) or with the humic and fulvic acids (HA, FA) from the regular and bioturbated soils. Cannella Valley, Majella massif, Italy

Treatment		*r*	*R* ^2^	df	*b*
GA		0.851**	0.933**	262	0.357
Regular soil					
Oe1	HA	0.877**	0.954*	142	0.099
	FA	n.s.	n.s.		n.s.
Oe2	HA	0.869**	0.919**	156	0.130
	FA	0.812**	0.914*	165	0.206
Bioturbated soil					
Straw tunnel wall (STW)	HA	n.s.	n.s.		n.s.
	FA	0.722**	0.872**	156	0.161
Vole feces (VF)	HA	0.892**	0.999*	180	0.182
	FA	0.847**	0.989*	190	0.112
Light tunnel wall (LTW)	HA	0.752*	0.836*	166	0.192
	FA	n.s.	n.s.		n.s.
Brown tunnel wall (BTW)	HA	0.815**	0.975*	186	0.162
	FA	n.s.	n.s.		n.s.
Upper‐outer layer of soil tunnel wall (UOSTW) (A2)	HA	n.s.	n.s.		n.s.
	FA	0.855**	0.949**	186	0.154
Upper‐inner layer of soil tunnel wall (UISTW) (A2)	HA	0.867**	0.976*	192	0.093
	FA	0.815**	0.936*	158	0.146
Lower‐inner layer of soil tunnel wall (LISTW) (A2′)	HA	n.s.	n.s.		n.s.
	FA	n.s.	n.s.		n.s.
Lower‐outer layer of soil tunnel wall (LOSTW) (A2′)	HA	n.s.	n.s.		n.s.
	FA	n.s.	n.s.		n.s.

df, degree of freedom; n.s., not significant, meaning that no significant coefficient of correlation and regression were found.

*P ≤ 0.05; **P ≤ 0.01; ***P = 0.001.

## Discussion

Morphology of the bioturbated soil differed from that of the regular one because of the convolution of horizons and galleries due to the snow vole burrowing activity. A similar situation was reported by Laundré and Reynolds (1993) and Yurkewycz et al. (2014), who found that burrowing of small mammals impacts a variety of soil processes including mineral distribution, aeration, and mineralization rates. It is worthy to note that the remainders of the snow tunnels hosted grass seedlings whose roots diffused into the materials itself in the case of LTW and BTW, while in the case of STW, roots penetrated the underlying soil horizons. The fact that seedlings were present in these materials but not in the adjacent soil indicated that seeds were part of the material used by the vole to line the tunnels and that the materials in which they were included represented a suitable micro‐environment for germination. In effect, these materials act as a natural mulching as the soft consistence favors the internal moisture conservation, while the dark color heats up the inside temperature. Depending on the year and species, both these factors may anticipate seed germination of 7–10 days with respect to the regular soil, thus evidencing the impact of the vole activity on the plant growing season in this harsh high‐mountain environment. Notwithstanding the calcareous nature of parent material and skeleton, both bioturbated and regular soils displayed an acidic reaction. This can be ascribed to the process of kettle hole filling and, for the bioturbated soil, to the snow vole activity. Once the kettle holes formed, because of their concave form they accumulated material washed in from the slopes or wind blown from other sources. Because of this, on the Majella massif, the filling particles had a texture ranging from sandy loam to clay loam (Corti et al. 2012) and consequently were easily weatherable because of the high specific surface. Thus, even if it contained limestone grains, during millennia the accumulated material produced superficial horizons with neutral to sub‐acid pHs. However, mineral horizons of regular soil never reached a pH lower than 5.7. As some sub‐superficial layer of the bioturbated soil showed a pH as low as 4.7–4.9, it is presumable that this acidification level was reached because of the burrowing and physiologic activity of the snow vole.

Regular soil was highly endowed of organic carbon, even though lower TEC/TOC percentages with respect to the soil tunnels walls and the A3 horizon of the bioturbated soil indicated that much of the SOM of the regular soil was poorly decomposed. In the bioturbated soil, the distribution of TOC, TEC, and humic substances suggested that each part of the vole home range represented a micro‐environments. For example, the relationship between TEC and TOC indicated the existence of a gradient of SOM decomposition ranging from very poor (STW) to relatively high (LISTW). Further, the higher percent of FA relative to TEC in the bioturbated than in regular soils suggested the occurrence of an early SOM humification (Sitzia et al. 2014) in the snow vole affected soil. This data show that the snow vole activity induces changes in SOM mineralization pathway and, consequently, the rates of nutrient cycling through decomposition and plant growth (Henry and Molau 1997). This ecological effect of the snow vole in mountain soils is similar to that of the gray‐tailed vole in agricultural soils, where SOM properties of the layers above the soil tunnel differ with respect to those of the layers below (Gervais et al. 2010).

Humic acids and FA from both bioturbated and regular soils differed for the distribution of their functional groups, and especially for carboxyl groups that abounded in all FA. In particular, the FA from STW contained the highest amount of carboxyl groups, peptides, and carbohydrates, as indicated by ^1^H HRMAS‐NMR and ^1^H‐^13^C HSQC spectrum data. The presence of easily degradable moieties in the FA structure, and the low content of aromatic groups in both HA and FA from STW and VF indicated their early stage of humification (Stevenson 1994). This result is expected in critical conditions such as those occurring in alpine environments where the lowering of mineralization and humification happens because of low temperatures, high humidity, and short period of biological activity (Budge et al. 2011). A continuous input of fresh organic matter due to vole activity further justify the presence of poorly humified material in the bioturbated soil. Our results agree with studies that defined the humic substances as a supramolecular association of heterogeneous molecules (sugar, fatty acids, polypeptides, aliphatic chains, and aromatic rings) held together by hydrophobic interactions (van der Waals, *π*‐*π*, ion‐dipole) and hydrogen bonds (Piccolo 2002). The presence of a well‐defined molecular structure (waxes, carbohydrates, and peptides) in the investigated HA and FA is a further support to supramolecular association. According to Nardi et al. (2009) and Canellas and Olivares (2014), poorly humified HA and FA may play a biological activity by exerting a positive influence on metabolic and signaling pathways involved in the plant development. Because of this, we expected that both HA and FA of our two types of soil could show a biological activity. Running the trials of hormone‐like activity with the humic substances of the regular soil, we found that the highest auxin‐like activity was shown by HA and FA from the Oe1 horizon, while the maximum gibberellin‐like activity was expressed by FA from the Oe2 horizon. These results showed the existence of a well‐distributed hormone‐like activity within the regular soil, as already reported for mountain forest soils by Pizzeghello et al. (2002). Indeed, HA and FA may regulate the development of root systems (Canellas and Olivares 2014) and the metabolism of sucrose available to plants growing cells (Nardi et al. 2000). The hormone‐like activity of the bioturbated soil was lesser than that of the regular soil, and this fact might be ascribed to the different composition of humic substances in terms of functional groups. The high presence of carboxylic acids, amides, proteins, and amino acids in the FA from STW and UISTW and in the HA from LTW might explain their IAA‐like activity, whereas the relative aromatic and amide components of both HA and FA from VF is supportive for their GA‐like activity. In fact, while carboxyl groups affect the auxin bioavailability (Napier 2004), aromatics and amides influence the gibberellins‐like activity (Vaughan and Malcolm 1985; Nardi et al. 2000). The hormone‐like activities of HA and FA from the bioturbated soil, although lower than those of the regular soil, are of high ecological significance because, together with the other soil changes induced by the vole activity (soil moisture and temperature, organic C, and nutrient cycling), contribute to make the snow vole home range a fertile hot‐spot within a poor and hostile environment. Indeed, the presence of vole, with respect to the regular soil, induces a major soil vertical complexity and a change in the pattern of SOM decomposition. The relative high presence of FA in the bioturbated soil confirms that snow vole induces for an early stage of humification. This was also supported by the fairly high amount in carboxylic groups, peptides, and carbohydrates found in the FA of the bioturbated soil, and aromatics in HA and FA of VF, which reflect in the hormonal behavior. Thus, FA and HA from the snow vole bioturbated soil act as natural amendments and stimulators for this prairie, as confirmed by the ample grass colonization of the abandoned snow vole home ranges.

At the high altitudes of the Majella Massifs (Italy), the soils inside kettle holes host dozens of snow vole colonies. The snow vole, with its winter snow tunneling and summer soil burrowing activity, stacks seeds into the materials used to line the tunnels and, after the snow melt, these seeds are forced to germinate because of the mulching effect exerted by the materials in which they are included. Burrowing activity also determines redistribution throughout the profile of SOM and hormone‐like activity. This activity stimulates seedlings to develop roots and supply energy to cells, which are two crucial processes to solve where, as in this case, the climatic conditions are harsh and the growing season is short. We speculate that, in this mountain cold environment, the burrowing activity of snow vole might have favored introduction and diffusion of plant species requiring a slightly longer growing season whose seeds would have been collected by the vole at lower altitudes. Doing this, the vole would anticipate climate warming and contribute to increase biodiversity.

## Data Accessibility

All data regarding the study site, field operations, and organic carbon content can be requested to Giuseppe Corti (g.corti@univpm.it), whereas the data set used for the regression analysis of the hormone‐like activity can be requested to Diego Pizzeghello (diego.pizzeghello@unipd.it). The curve fitting procedure applied to the investigated region of the ATR/FTIR spectra is reported as supporting information. Questions on the ATR/FTIR spectra can be requested to Ornella Francioso (ornella.francioso@unibo.it) and for the ^1^H HRMAS‐NMR spectra to Erika Ferrari (erika.ferrari@unimore.it).

## Conflict of Interest

None declared.

## Supporting information


**Appendix S1** ATR/FTIR spectra Gaussian curve fitting.
**Figure S1** Histograms of HA (upper) and FA (lower) of the ATR/FTIR peak areas processed by using Gaussian curve fitting.
