# Supplementary material for: Snow vole (Chionomys nivalis Martins) affects the redistribution of soil organic matter and hormone‐like activity in the alpine ecosystem: ecological implications
Source: Ecol Evol. 2015 Sep 29;5(20):4542–54. doi: 10.1002/ece3.1727 (PMC4670049; doi:10.1002/ece3.1727)
Supplement: Supplementary file 1 — Appendix S1 ATR/FTIR spectra Gaussian curve fitting. Figure S1 Histograms of HA (upper) and FA (lower) of the ATR/FTIR peak areas processed by using Gaussian curve fitting. [file ECE3-5-4542-s001.docx]

**Snow vole (Chionomys nivalis Martins) affects the redistribution of soil organic matter and hormone-like activity in the alpine ecosystem: ecological implications**

Diego Pizzeghello^*,1^, Stefania Cocco^2^, Ornella Francioso^3^, Erika Ferrari^4^, Alessandra Cardinali^1^, Serenella Nardi^1^, Alberto Agnelli^5^ and Giuseppe Corti^2^

^1^Dipartimento di Agronomia, Animali, Alimenti, Risorse Naturali e Ambiente (DAFNAE), Università di Padova, Legnaro, Padova, Italy

^2^Dipartimento di Scienze Agrarie, Alimentari e Ambientali, Università Politecnica delle Marche, Ancona, Italy

^3^Dipartimento di Scienze Agrarie, Università di Bologna, Bologna, Italy

^4^Dipartimento di Scienze Chimiche e Geologiche, Università di Modena e Reggio Emilia, Modena, Italy

^5^Dipartimento di Scienze Agrarie, Alimentari ed Ambientali, Università di Perugia, Perugia, Italy

^*^ Corresponding author: diego.pizzeghello@unipd.it

**Supporting information**

**ATR/FTIR spectra Gaussian curve fitting**

More quantitative information on the ATR/FTIR spectra of HA and FA were obtained by using Gaussian curve fitting procedure applied to the investigated region. The optimum curve-fitting was determined by the lowest value for the minimization function of reduced Chi square (χ2) and a good agreement between experimental and calculated profiles [coefficients of determination (R2) between 0.999 and 0.988, and the standard error (SE) in the range of 0.001-0.003]. Thus, the percentage area of each functional group can be considered representative of the HA and FA structures (Supporting information Fig. 1 upper and lower, respectively). Carboxylate groups represented the most abundant functional groups in HA. These groups accounted for 70% in the HA of UOSTW, 65% in UISTW, 60% in Oe1 and Oe2 horizons, 56 in LTW, 55% in STW and 49% in BTW. In contrast, carboxylate groups in FA from regular soil accounted for 48 and 40% in Oe1 and Oe2, respectively. Carbonyl group (COOH) in HA accounted for 5.4% in UOSTW, 2.5% and 1.7% in Oe1 and Oe2, respectively. These groups were more abundant in FA, where they accounted for 28% in UISTW, 26% in STW, 20% in BTW, 19 and 18% in UOSTW and LTW, respectively. The presence of amide I was the most representative in FA bioturbated soil accounting for 55% in UISTW and UOSTW, 53% in STW, LTW and BTW. Amide I was absent in FA of the regular soil. Aromatic rings were the second functional group that characterized HA; they accounted for 19% in LTW, 15% in UOSTW, 13% in UISTW, 12% in STW, 11% in BTW, 9.9 and 6% in Oe1 and Oe2, respectively. In FA, the aromatic component might be influenced by the amide II contribution and, for this reason, we have considered all together the percentage area: 14% in Oe1, 13% in LTW, 12% in UOSTW, 11% in BTW, 10% in UISTW, 9% in Oe2, and 8% in STW. A minor influence was due to aliphatic groups (CH_3_), which in HA accounted for 16% in LTW, and for 9 and 7% in Oe1 and Oe2, respectively, 1.3% in UOSTW, 1% in both BTW and UISTW, 0.9% in STW. In FA they accounted for 13% in Oe2, 7.6% in STW, 6% in UISTW, 4% in LTW, BTW and UOSTW. Similarly, C-OH groups in HA accounted for 10% in BTW, 6% in STW and 2.1% in LTW; 7 and 5% in Oe2 and Oe1, respectively. In FA they accounted from 4% in both LTW and BTW, and 3.5 % in UOSTW.

**Figure captions**

**Supporting information Figure 1.** Histograms of HA (upper) and FA (lower) of the ATR/FTIR peak areas processed by using Gaussian curve fitting. The optimum curve-fitting was determined by the lowest value for the minimization function of reduced Chi square (χ2) and a good agreement between experimental and calculated profiles [coefficients of determination (R2) between 0.999 and 0.988 and the standard error (SE) in the range of 0.001-0.003].
